# New strategy for intraoperative phonosurgical management of recurrent laryngeal nerve infiltrated by thyroid carcinoma

**DOI:** 10.1007/s00423-024-03323-x

**Published:** 2024-04-27

**Authors:** Jieying Peng, Guanghao Zhu, Yingna Gao, Xianmin Song, Haojun Yu, Rushi Huang, Mengjie Chen, Yafei Jiang, Guodong Sun, Meng Li, Hongliang Zheng, Wei Wang

**Affiliations:** 1 Department of Otolaryngology-Head and Neck Surgery, The First Affiliated Hospital of Naval Medical University, Shanghai, China; 2 Shanghai 411 hospital, Shanghai, China; 3 Hangzhou Special Service Recovery Center of Air Force, Hangzhou, China

**Keywords:** Recurrent laryngeal nerve, Vocal fold paralysis, Laryngeal reinnervation, Ansa cervicalis, Arytenoid adduction, Thyroid carcinoma

## Abstract

**Purpose:**

Treating an infiltration of the recurrent laryngeal nerve (RLN) by thyroid carcinoma remains a subject of ongoing debate. Therefore, this study aims to provide a novel strategy for intraoperative phenosurgical management of RLN infiltrated by thyroid carcinoma.

**Methods:**

Forty-two patients with thyroid carcinoma infiltrating the RLN were recruited for this study and divided into three groups. Group A comprised six individuals with medullary thyroid cancer who underwent RLN resection and arytenoid adduction. Group B consisted of 29 differentiated thyroid cancer (DTC)patients who underwent RLN resection and ansa cervicalis (ACN)-to-RLN anastomosis. Group C included seven patients whose RLN was preserved.

**Results:**

The videostroboscopic analysis and voice assessment collectively indicated substantial improvements in voice quality for patients in Groups A and B one year post-surgery. Additionally, the shaving technique maintained a normal or near-normal voice in Group C one year post-surgery.

**Conclusion:**

The new intraoperative phonosurgical strategy is as follows: Resection of the affected RLN and arytenoid adduction is required in cases of medullary or anaplastic carcinoma, regardless of preoperative RLN function. Suppose RLN is found infiltrated by well-differentiated thyroid cancer (WDTC) during surgery, and the RLN is preoperatively paralyzed, we recommend performing resection the involved RLN and ACN-to-RLN anastomosis immediately during surgery. If vocal folds exhibit normal mobility preoperatively, the MACIS scoring system is used to assess patient risk stratification. When the MACIS score > 6.99, resection of the involved RLN and immediate ACN-to-RLN anastomosis were performed. RLN preservation was limited to patients with MACIS scores ≤ 6.99.

**Supplementary Information:**

The online version contains supplementary material available at 10.1007/s00423-024-03323-x.

## Introduction

Thyroid carcinoma ranks as one of the most prevalent endocrine malignancies. Over recent decades, the incidence of thyroid cancer has increased rapidly in several countries, including China [1]. Extrathyroidal tumor extension is one of the most crucial adverse prognostic factors in thyroid cancer [2]. Among patients with papillary thyroid cancer (PTC) who have extrathyroidal invasion, the rate of RLN involvement is 33–61% [3, 4]. The RLN is one of the most commonly affected structures in cases of locally invasive thyroid cancer due to its anatomical proximity to the thyroid gland. In cases where the cancer clearly infiltrates the nerve, surgeons face a decision: whether to sacrifice the nerve or release it from its restrictions to preserve its function. Some surgeons opt to sacrifice the affected RLN during the excision, regardless of its preoperative function [5, 6]. Conversely, other studies have indicated that achieving complete excision through resection of the RLN does not lead to improved survival when DTC has infiltrated the nerve. These studies advocate for a preservation approach, with maximum effort to remove all macroscopically apparent tumor portions around the nerve [7, 8]. However, these studies had limitations, including a brief follow-up period, small number of cases, or non-homogeneous distribution of extrathyroidal involvement among the compared groups. While preserving the RLN that is affected by cancer may offer some functional advantages, concerns persist regarding its impacts on surgical thoroughness and oncological safety. Furthermore, Surgeons continue to have concerns regarding the optimal management of severe RLN in cases were sacrificing the affected RLN becomes necessary.

**Fig. 1:**
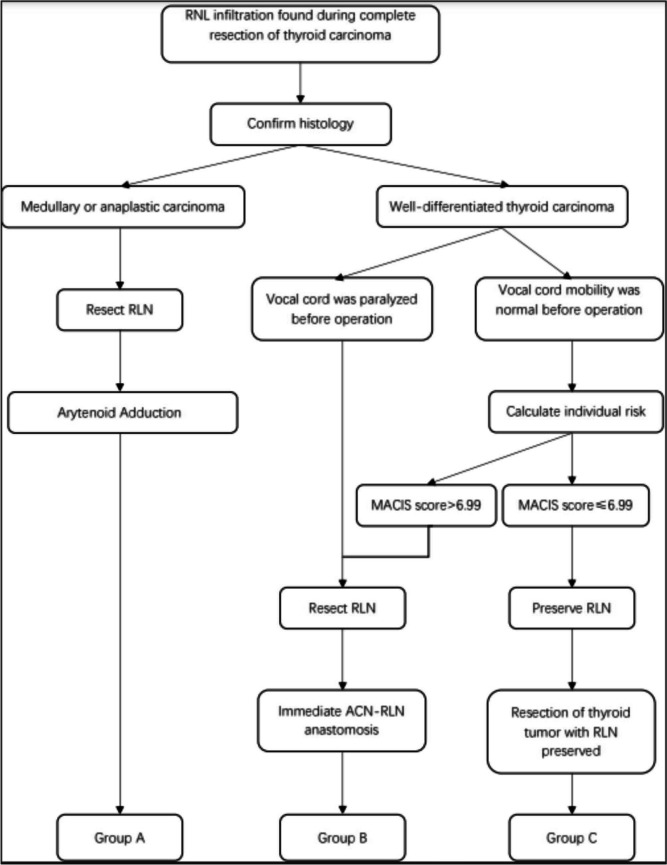
Flow chart of patient enrollment and classification

The 2018 the American Head and Neck Society consensus statement for managing the RLN during thyroidectomy suggests resection of the RLN if it is nonfunctioning due to invasion; and preserving the nerve if the invaded RLN remains functional, shaving or partial excision of the nerve sheath for superficial invasions; For cases of more extensive invasion, it is advisable to conduct intraoperative nerve monitoring to assess the functional status of the recurrent laryngeal nerve [7, 9]. In China and most of developing countries, due to medical insurance coverage limits, neuromonitoring is not routinely performed in every thyroid surgery. Consequently, we need a decision-making strategy that contains the assessment of thyroid cancer prognosis, which could assist in determining the appropriate approach to deal with the condition of recurrent laryngeal nerve invaded by the tumor. The metastases, age, completeness of excision, invasion, and size (MACIS) classification serve as parameters for assessing the completeness of surgical resection, tumor invasiveness, and risk stratification for patients with DTC [10, 11]. Boucek et al. reported a surgical strategy for managing RLN infiltrated by well-differentiated thyroid cancer (WDTC) based on the MACIS system [12]. Moreover, there is no decision-making strategy for the intraoperative phonosurgical management of RLN infiltrated by thyroid carcinoma. Phonosurgical treatment options for unilateral vocal cord paralysis (UVCP) resulting from RLN injury encompasses vocal cord injections, medialization thyroplasty, arytenoid adduction, and laryngeal reinnervation [13, 14]. All the above phonosurgical treatments were performed in our clinical practice. Drawing upon our phonosurgical expertise and Boucek’s strategy, this study aims to introduce a decision-making strategy for the intraoperative phonosurgical management of RLN infiltrated by thyroid carcinoma. Several factors are considered in this strategy, including thyroid carcinoma pathologies, preoperative RLN function, and MACIS classification. (Fig. 1).

## Materials and methods

### Patient enrollment

The Institutional Review Board of the Naval Medical University approved this study. Our study is a retrospective study. The medical records of 47 patients whose RLN were invaded by thyroid carcinoma between January 2005 and December 2021 were reviewed. The follow-up period for all patients was 1-year postoperatively. Five patients who were lost to follow-up were excluded from the study. Finally, 42 patients whose RLN was invaded by thyroid carcinoma were recruited. Among them, six patients diagnosed with medullary carcinoma underwent resection of the affected RLN and arytenoid adduction for phonosurgical treatment. They made up Group A. Twenty-nine patients with DTC who underwent resection of the affected RLN along with ACN-to-RLN anastomoses were included in Group B. In Group B, there were 19 patients categorized under the fixed vocal cord subgroup, all of whom exhibited UVCP before the operation. Additionally, the remaining 10 patients in Group B presented with preoperative normal vocal cord mobility, and their MACIS scores exceeded 6.99. They were classified as mobile vocal cord subgroup. Seven patients had their RLNs invaded by DTC, and their RLNs were normal before the operation. Their MACIS scores were ≤ 6.99. In these cases, every effort was made to preserve the RLNs while ensuring the removal of all macroscopically visible tumor portions around the nerve. Subsequently, seven patients were included in Group C (Fig. 1).

Videostroboscopy and voice assessment were performed on all groups before and 1 year after surgery. However, the mobile vocal cord subgroup of Group B underwent the above examinations one month and one year after surgery to assess the effectiveness of the ACN-to-RLN anastomosis. This additional assessment was conducted due to the normal mobility of the vocal cord observed in the preoperative state of cases within the mobile vocal cord subgroup. The assessment of subjective aspiration rating was performed both before and three months after the operation. Additionally, various examinations were conducted to confirm the size, and extent of invasion of the thyroid tumor.

### Surgical procedure

#### Arytenoid adduction procedure

**Fig. 2:**
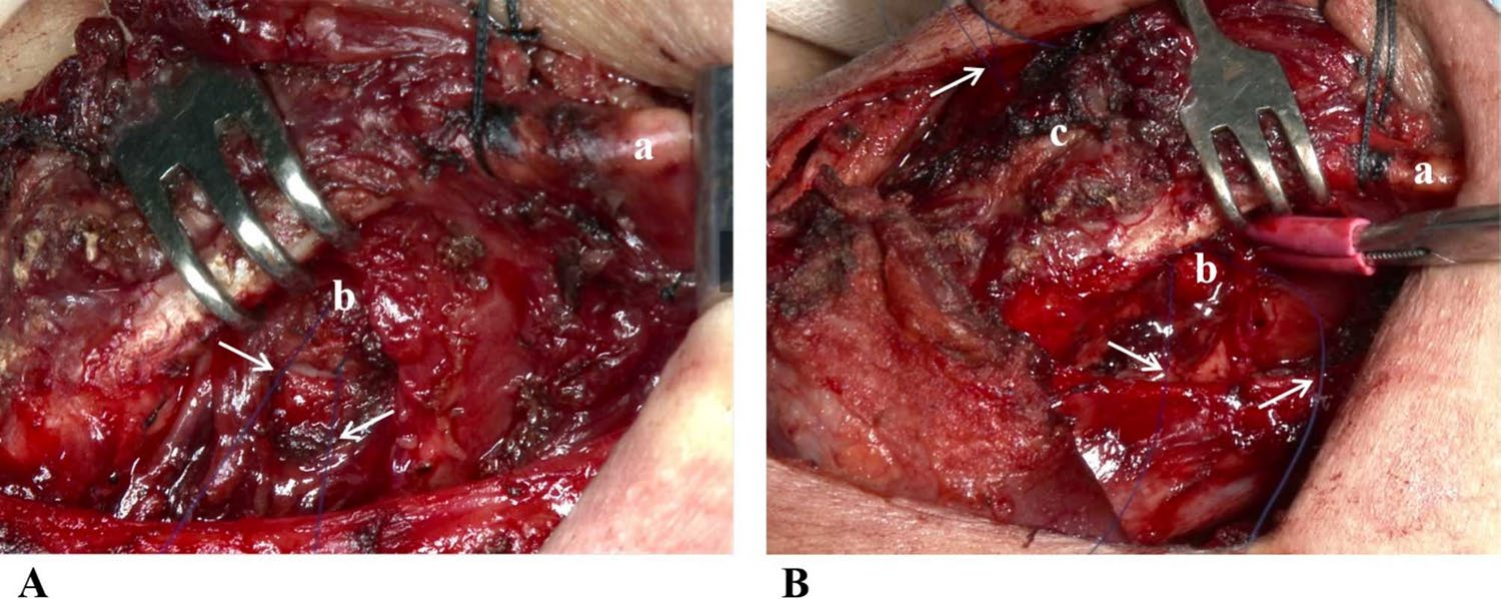
Intraoperative picture of arytenoid adduction procedure. A shows that two 5–0 prolene sutures were passed through the muscular processes of arytenoid cartilage. B shows that two sutures were parallel to the middle and lower 1/3 level of the thyroid cartilage. The two-suture tension was adjusted to rotate the vocal process of the arytenoid cartilage medially, consequently adducting the true vocal fold. a: Superior cornu of thyroid cartilage; b: Arytenoid cartilage; c: Thyroid cartilage; Arrow:5–0 prolene sutures

Our previous study reported an arytenoid adduction, following the method described by Isshiki et al., with minor modifications [15]. This technique was used in Group A and was briefly summarized as follows: The inferior pharyngeal constrictor muscle was incised along the lateral edge of the thyroid cartilage. After delicately separating the mucosa of the piriform sinus the capsule of the cricoarytenoid joint was incised and exposed. Two 5–0 prolene sutures were then passed through the muscular processes of arytenoid cartilage (Fig. 2A). The sutures were parallel to the middle and lower 1/3 level of the thyroid cartilage. Their ends were passed through two holes created using a 16-gauge stitch. The two-suture tension was adjusted to rotate the vocal process of the arytenoid cartilage medially, consequently adducting the true vocal fold to realign the arytenoid closer to the physiological adduction position (Fig. 2B).

#### ACN -to-RLN anastomosis procedure

**Fig. 3:**
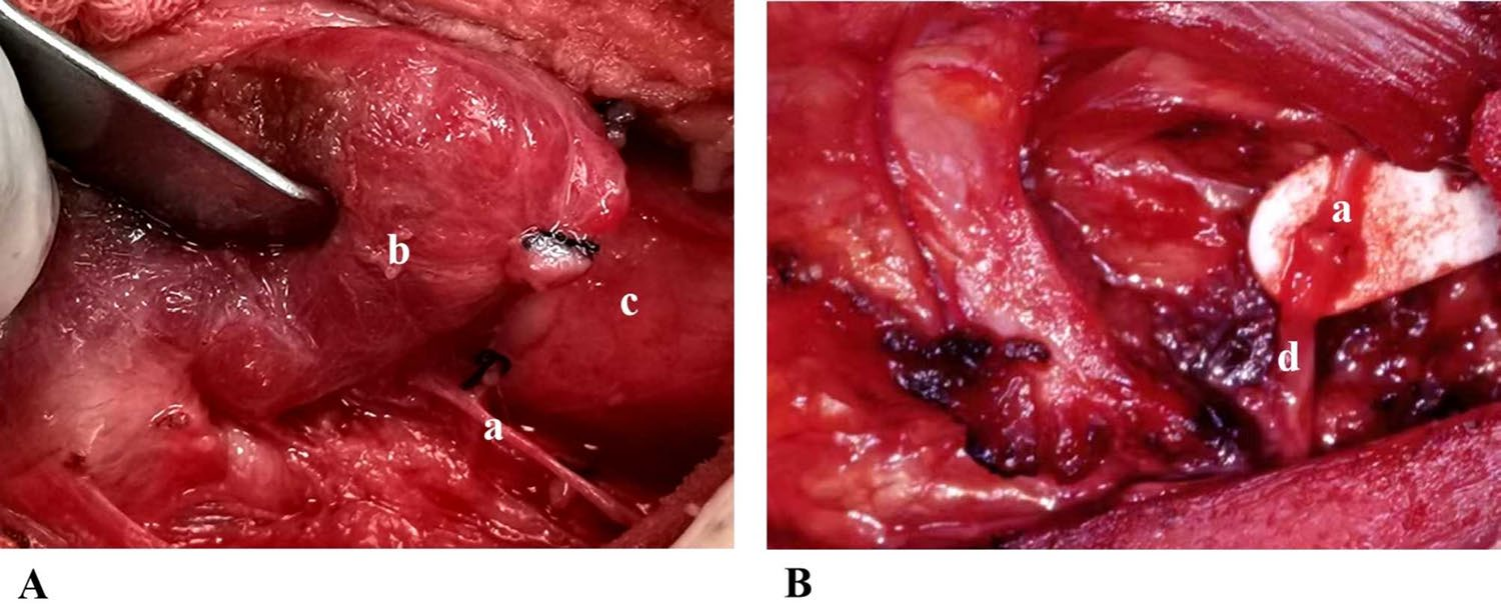
Intraoperative picture of resecting the invaded RLN and ACN-to-RLN anastomosis. A shows that the thyroid tumor was invated the right RLN. The distal stump of the intrapharyngeal RLN was located behind the thyroid cartilage. B shows that after total thyroidectomy and central cervical lymph nodes dis-section as well as the invaded right RLN resection, the distal stump of the right RLN was anastomosed to the main branch of ipsilateral ansa cervicalis. a: RLN; b: Thyroid tumor; c: Trachea; d: Ansa cervicalis

The ACN-to-RLN anastomosis procedure used in Group B was previously described in our earlier study [16]. This is briefly explained as follows: The ACN loop is typically identified through fascia, which runs over the common carotid artery or the jugular vein. The main branch of ACN was truncated at the bifurcation point and made ready for anastomosis. The distal stump of the intrapharyngeal RLN was located behind the thyroid cartilage(Fig. 3A). The distal end of the RLN was tension-free and anastomosed to the main branch of the ACN using three to five epineural stitches of 11–0 nylon thread under an operating microscope. In cases where the ACN lacks a trunk or two or three branches, the anterior roots of the ACN were fitted to the distal stump of the RLN(Fig. 3B) [17].

#### Preserving RLN with shaving technique

**Fig. 4:**
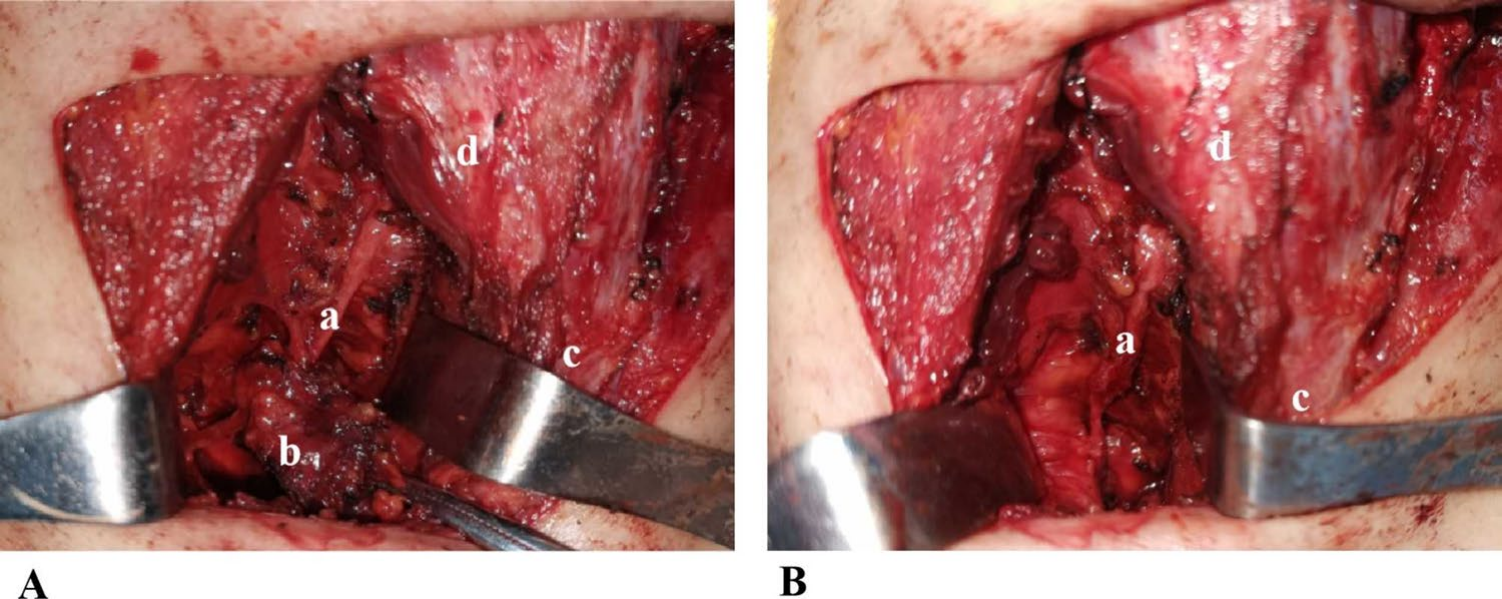
Intraoperative picture of preserving RLN with shaving technique. A shows that the right RLN was invaded by the thyroid tumor. B shows that the affected RLN was delicately peeled off from the tumor with a combination of blunt and precise dissection. a: RLN; b: Thyroid tumor; c: Trachea; d: Thyroid cartilage

In Group C, the shaving technique was employed to preserve the RLN. To achieve RLN preservation, the affected RLN was delicately peeled off from the tumor with a combination of blunt and precise dissection (Fig. 4A). The nerve preservation strategy involved excising as much of the tumor as possible while taking precautions to avoid nerve injury. These procedures were performed under an operating microscope, utilizing the shaving technique (Fig. 4B).

### Videostroboscopy

Using a videostroboscope (Wolf, model 5570), all patients were observed while producing the sound “eee” at a comfortable loudness and pitch for as long as possible. Dynamic videos of the phonation were recorded. Subsequently, three experienced laryngologists who were blinded to the procedures assessed the recorded videos. The videos were randomized, and the reviewers were blinded to whether they were recorded pre- or post-surgery. The visual laryngeal analysis focused on assessing glottal closure (0 = complete; 1 = slightly incomplete; 2 = moderately incomplete; and 3 = severely incomplete). The reviewers reached a consensus on the visual evaluation of the larynx.

### Voice assessment

Voice assessment included perceptual evaluation, acoustic analysis, and maximum phonation time (MPT). Speech samples containing sustained vowels /a/ and connected-speech samples were subjected to perceptual evaluation and acoustic analysis. The recording setup included a digital recorder and dynamic microphone (Tiger Electronics Inc., North Reading, MA, USA). Five laryngologists used the perceptual rating scale (GRBAS) to assess speech quality and features. The ratings were performed in a blinded manner, with patient speech samples arranged randomly. The listeners were instructed to assess the segmented speech samples, providing ratings for overall grade. Each listener used a perception scale to rate the sound quality of overall grade (0 = normal, 1 = mild, 2 = moderate, and 3 = severe). The average values were computed across all five listeners.

The acoustic parameters of the sustained vowel /a/ were evaluated using the Praat software (Boersma et al., David (2011). Praat: Doing phonetics using a computer [computer program]. Version 5.1.12; retrieved from http://www.praat.org/). The acoustic parameters encompassed the mean noise-to-harmonics ratio (NHR) and measures of phonatory stability, specifically jitter (local) and shimmer (local). Additionally, maximum phonation time (MPT), which signifies the duration of sustained phonation of the vowel /a/ following maximum inspiration, was measured before and after surgery. The MPT is generally considered an indicator of glottis efficiency.

### Subjective aspiration rating

Aspiration was assessed subjectively both before and three months after the operation using a four-point scale as described by Cummings [18]. The severity of aspiration was rated on a scale as follows: no aspiration = 0, mild = 1, moderate = 2, and severe = 3. Aspiration was considered mild when there was occasional choking but did not require dietary modification. Moderate aspiration was associated with symptoms necessitating dietary modifications. Aspiration was considered severe when patients required feeding tube placement to prevent pulmonary complications associated with aspiration [15].

### Statistical analyses

The jitter (local), shimmer (local), NHR, and MPT data exhibited a non-normal distribution and were presented as medians (low and upper quartile). The differences between the pre- and postoperative glottal closure, overall grade, jitter (local), shimmer (local), NHR, and MPT were evaluated using the Wilcoxon signed-rank test. Differences in the postoperative data for parameters mentioned in Groups A, B, and C were evaluated using Kruskal–Wallis one-way ANOVA (all pairwise methods were used in multiple comparisons). Inter-rater and intra-rater reliabilities in the perceptual evaluation were analyzed using kappa coefficients. Statistical analysis was conducted using the SPSS software (version 25.0; SPSS Inc., Chicago, IL, USA). Statistical significance was set at *p* < 0.05.

## Results

### Patient characteristics

This study included 28 patients with papillary carcinoma, eight with follicular carcinoma, and six with medullary carcinoma. In Group A, there were 2 males and 4 females, and the mean age was 40.4 years old, age range was 38–75 years. In Group B, there were here were 6 males and 23 females, and the mean age was 44.8 years old, age range was 34–52 years. In Group C, there were 2 males and 5 females, and the mean age was 30.5 years old, age range was 29–34 years. All patients with DTC were treated with radioactive iodine and thyroid-stimulating hormone suppression using exogenous thyroxine postoperatively.

### Videostroboscopic findings

Preoperative video recordings showed moderately to severely incomplete glottal closure in most patients of Groups A and B, without significant difference between the groups (*p* > 0.05). One-year post-operation, both groups exhibited significant improvements in glottal closure. Group C had normal vocal cord mobility before surgery, with 57.1% preserving RLN function post-surgery. Postoperative improvement in glottal closure was significant in Groups A and B (*p* < 0.05) but not in Group C (*p* > 0.05). Significant differences in postoperative glottal closure were noted between Groups A and B and A and C (*p* < 0.05), but not between B and C (*p* > 0.05, refer to Table 1).

**Table 1:** Comparison between Preoperative and Postoperative Videostroboscopic Findings in Groups A, B, and C, and intergroup comparison of the three groups

	Groups	Changes Between Preoperative and Postoperative Values			*P* Values of Preoperative and Postoperative Comparison	*P* Values of Intergroups Comparison of Postoperative Values
		*N*	Preoperative Median (Q_L_, Q_U_)	Postoperative Median (Q_L_, Q_U_)		
Glottal Closure	A	6	2.5 (2, 3)	1 (0.75, 1.25)	*P* = 0.024	*P* total < 0.001, *P* ab < 0.001, *P* bc = 0.268, *P* ac = 0.016
	B	29	2 (2, 3)	0 (0, 0)	*P* < 0.001	
	C	7	0 (0, 0)	0 (0, 0)	*P* = 0.317	

### Voice assessment

#### Perceptual evaluation

Table 2 shows Groups A and B showed significant improvement in overall grades after the procedure (*P* < 0.05). Group C had normal or near-normal preoperative overall grades, and there was no significant difference between the pre- and postoperative overall gradess (*P* > 0.05). The postoperative overall grades of Groups B and C were significantly better than those of Group A (*P* < 0.05), but there was no significant difference between Groups B and C (*P* > 0.05). The inter-rater and intra-rater reliability of five listeners was acceptable (inter-rater reliability > 0.76, intra-rater reliability > 0.81).

**Table 2:** Comparison between preoperative and postoperative values of overall grade in Groups A, B, and C, and intergroup comparison of the three groups

	Groups	Changes Between Preoperative and Postoperative Values			*P* Values of Preoperative and Postoperative Comparison	*P* Values of Intergroups Comparison of Postoperative Values
		*n*	Preoperative Median(Q_L_, Q_U_)	Postoperative Median(Q_L_, Q_U_)		
Overall Grade	A	6	2.4 (2.35, 3)	1.4 (0.65, 1.85)	*P* = 0.027	*P* total = 0.002, *P* ab < 0.001, *P* bc = 0.156, *P* ac = 0.037
	B	29	2.2(2.2, 2.6)	0.2 (0, 0.4)	*P* < 0.001	
	C	7	0 (0, 0.4)	0.4 (0, 0.8)	*P* = 0.083	

#### Acoustic analysis

Postoperative values of jitter (local), shimmer (local), and NHR showed significant improvement than that in preoperative values in Groups A (*p* < 0.05, respectively) and B (*p* < 0.001, respectively) (see Table 3). Moreover, the postoperative values of these parameters exhibited no significant differences than in the preoperative values in Group C (*p* > 0.05) (see Table 3). Further analysis revealed no significant differences in the preoperative values of these parameters between Groups A and B (*p* > 0.05). Moreover, the postoperative values of these parameters in Group B were significantly lower than those in Group A (*p* < 0.05), while no significant differences were observed when compared to those in Group C (*p* > 0.05). The postoperative values of the above parameters in Group A were significantly higher than in those in Group C (*p* < 0.001) (Table 3).

**Table 3:** Comparison between preoperative and postoperative acoustic analysis in groups A, B, and C, and intergroup comparison of the three groups

Parameters	Groups	Changes Between Preoperative and Postoperative Values			*P* Values of Preoperative and Postoperative Comparison	*P* Values of Intergroups Comparison of Postoperative Values
		*n*	Preoperative Median (Q_L_, Q_U_)	Postoperative Median (Q_L_, Q_U_)		
Jitter Local (%)	A	6	1.40(1.28, 3.01)	0.70 (0.56, 0.82)	*P* = 0.028	*P* total < 0.001, *P* ab < 0.001, *P* bc = 0.105, *P* ac = 0.003
	B	29	1.39(1.02, 2.15)	0.24 (0.19, 0.34)	*P* < 0.001	
	C	7	0.21 (0.14, 0.25)	0.20 (0.15, 0.21)	*P* = 0.866	
Shimmer local (%)	A	6	10.16 (6.96, 12.67)	5.59 (4.46, 7.26)	*P* = 0.028	*P* total = 0.017 *P* ab = 0.015, *P* bc = 0.548, *P* ac = 0.003
	B	29	7.31(6.26, 8.58)	2.99(1.47, 5.32)	*P* < 0.001	
	C	7	2.71(1.87,2.87)	2.47 (1.99, 3.59)	*P* = 0.866	
NHR (-dB)	A	6	0.145(0.138, 0.175)	0.080 (0.060, 0.105)	*P* = 0.027	*P* total = 0.002, *P* ab < 0.001, *P* bc = 0.566, *P* ac = 0.008
	B	29	0.110(0.090, 0.145)	0.040 (0.030, 0.050)	*P* < 0.001	
	C	7	0.030(0.020, 0.070)	0.050 (0.020, 0.050)	*P* = 1.000	
MPT (second)	A	6	6.30(4.84, 7.02)	14.48 (13.29, 16.30)	*P* = 0.028	
	B	29	7.01 (5.24, 7.85)	18.98 (16.45, 22.46)	*P* < 0.001	*P* total = 0.003, *P* ab = 0.001, *P* bc = 0.484 *P* ac = 0.007
	C	7	19.68 (18.28, 24.47)	19.97 (16.48, 23.23)	*P* = 0.310	

#### MPT

MPT is generally considered an indicator of glottal efficiency. There was no significant difference in preoperative MPT values between Groups A and B (*p* > 0.05). However, the postoperative MPT value significantly exceeded the preoperative values in Groups A (*p* = 0.028) and B (*p* < 0.001) (Table 3). Furthermore, the postoperative MPT value in Group C showed no significant difference from the preoperative value (*p* > 0.05). Further analysis revealed that the postoperative MPT in Group B was significantly greater than that in Group A (*p* = 0.001). However, no significant difference was observed compared to that in Group C (*p* > 0.05). Postoperative MPT values in Group C were significantly higher than those in Group A (*p* < 0.001).

### Subjective aspiration rating (SAR)

Immediately after surgery, Group A patients showed improved aspiration symptoms due to arytenoid adduction (*p* = 0.024, Table 4). ACN-to-RLN anastomosis improved aspiration three months post-surgery (*p* < 0.001, Table 4). The shaving technique preserved swallowing function, with only one patient experiencing mild postoperative aspiration (*p* > 0.05, Table 4). No significant differences in postoperative SAR scores were found among the three groups three months post-surgery (*p* > 0.05, Table 4).

**Table 4:** Comparison between preoperative and postoperative values of subjective aspiration rating in groups A, B, and C, and intergroup comparison of the three groups

Parameters	Groups	Changes Between Preoperative and Postoperative Values			*P* Values of Preoperative and Postoperative Comparison	*P* Values of Intergroups Comparison of Postoperative Values
		*n*	Preoperative Median (Q_L_, Q_U_)	Postoperative Median (Q_L_, Q_U_)		
SAR	A	6	2 (1, 2)	0 (0, 0.25)	*P* = 0.024	*P* total = 0.918, *P* ab = 0.825, *P* bc = 0.705 *P* ac = 0. 909
	B	29	2 (1, 2)	0 (0, 0)	*P* < 0.001	
	C	7	0 (0, 0)	0 (0, 0)	*P* = 0.317	

QL, low quartile; QU upper quartile. SAR, Subjective Aspiration Rating

## Discussion

Preserving or restoring laryngeal function should be a key consideration when dealing with RLN involvement in thyroid carcinoma. The MACIS system was established by Hay et al. in 1993 [11]. The MACIS scoring system considers metastasis, age, completeness of resection, local invasion, and tumor size. In the MACIS scoring system, a score is obtained using a mathematical formula to predict cause-specific survival (CSS) in patients with DTC. The patients were categorized into one of three groups based on their MACIS score, which helps predict their probability of survival (see Table S1, cited from Hay [11] and McCaffrey [19]).

In our new strategy, the decision to either preserve or resect the affected RLN is determined by factors such as the pathology of the thyroid carcinoma, preoperative RLN function, and MACIS score. First, if the preoperative FNAB or intraoperative frozen section indicates that the histology of the thyroid cancer is medullary carcinoma, resection of the involved RLN is needed, regardless of the preoperative RLN function. All patients in Group A had medullary thyroid carcinoma, and the one-year survival rate for this type of carcinoma was 50% [20], which was significantly lower than that observed in cases of DTC. Therefore, arytenoid adduction was recommended for this Group. The median survival time from the diagnosis of anaplastic thyroid carcinoma is approximately 5.3 months, with a 1-year survival rate of approximately 29% [21, 22]. In our clinical study, we encountered several cases of anaplastic thyroid carcinoma invading the RLN. However, because the cancer had already invaded the larynx and/or trachea, we surgically removed the larynx and trachea, making voice reconstruction impossible. Therefore, we recommend performing arytenoid adduction concurrently with the thyroidectomy. This approach can lead to immediate improvements in post-operative aspiration and dysphonia, significantly enhancing the quality of life of patients. However, ACN-to-RLN anastomosis is not a suitable option for patients with RLN infiltration by medullary or anaplastic thyroid carcinoma because of their short life expectancy. The process of regenerative nerve fiber reinnervation for the laryngeal muscles can be relatively time-consuming. Second, suppose the RLN is pre-operatively paralyzed and is found to be infiltrated by DTC during surgery. In that case, we recommend combining en bloc resection of the thyroid carcinoma involving the RLN with an ACN-to-RLN anastomosis performed immediately during the operation. This recommendation is based on the understanding that even if the surgeon preserves the RLN in such cases, the paralyzed vocal fold will not regain its mobility. Third, suppose vocal folds exhibit normal mobility before surgery, and the RLN is found to be infiltrated by WDTC during the operation. In that case, the MACIS scoring system is used to assess the risk stratification for the individual patient. If the MACIS score of the patient exceeds 6.99 and their CSS drops below 89%, complete resection of the tumor with the involved RLN should be required. In such instances, immediate ACN-to-RLN anastomosis should be performed during thyroid cancer extirpation. (see Fig. 1).

Our previous large-scale study and other reports have demonstrated that ACN-to-RLN anastomosis is among the most widely utilized procedures for laryngeal reinnervation [17, 23, 24]. Our present study indicated that in cases where the RLN is infiltrated by WDTC, conducting an immediate ACN-to-RLN anastomosis during the complete excision of the DTC could restore satisfactory phonatory function without compromising oncological radicality. Furthermore, the post-operative videostroboscopy and voice assessment outcomes indicated that the voice quality of patients who underwent immediate ACN-to-RLN anastomosis was better than that of patients who underwent arytenoid adduction. Patients with WDTC experience a more favorable 10-year survival rate, allowing ansa nerve fibers sufficient time to regenerate and restore the mass and tension of the paralyzed vocal folds within the laryngeal muscles. The regenerated nerve fibers stemming from the ACN extended along the endoneurial tube of the RLN, eventually innervating the laryngeal muscles; however, the laryngeal adductor muscles were stronger than the abductor muscles. The reinnervated vocal folds are usually fixed at or near the midline, resulting in complete glottic closure postoperatively.

Additionally, preservation of the RLN using the shaving technique has been reported by Randolph, et al. [7, 8]. Their studies revealed no prognostic differences between complete excision of the affected nerve and preservation of the nerve while performing macroscopic radical removal of the tumor tissue. In the present study, if RLN function was normal pre-operatively and WDTC infiltration was discovered during operation and provided the MACIS score was ≤ 6.99, we recommend considering the preservation of the RLN using the shaving technique during the operation to balance the oncological safety and phonatory function of patients (see Fig. 1). Preservation of the RLN, which results in incomplete excision of the WDTC, should be followed by post-operative radioactive iodine and TSH suppression. The present study indicated that the shaving technique could preserve a normal or near-normal voice in Group C 1 year after surgery.

Limitations of our study includes the retrospective study design and the referral bias, characterized by the transfer of complex cases to tertiary centers, results in diverse baseline characteristics. Our researchers recognize that further research will enroll more cases, and would be powered to detect statistically significant differences in 10-year recurrence-free survival and 10-year overall survival.

## Conclusions

Based on our phonosurgical experience and strategy from Boucek, we introduce a decision-making strategy for the intra-operative phono-surgical management of RLN infiltrated by thyroid carcinoma. This strategy takes into account various factors, including the type of thyroid carcinoma, preoperative RLN function, and MACIS classification. Specifically, suppose the histological analysis indicates that the thyroid cancer is of medullary or anaplastic carcinoma. In that case, the standard protocol involves the resection of the affected RLN, regardless of preoperative RLN function. During thyroidectomy, we recommend immediate arytenoid adduction. Suppose the RLN is pre-operatively paralyzed and is found to be infiltrated by WDTC during surgery. In that case, we recommend that en bloc resection of the thyroid carcinoma with the involved RLN should be combined with ACN-to-RLN anastomosis immediately during surgery. However, when vocal folds exhibit normal mobility pre-operatively, and the RLN is found to be infiltrated by WDTC during operation, we employ the MACIS scoring system to assess the risk profile of the patient. When the MACIS score was > 6.99, resection of the involved RLN and immediate ACN-to-RLN anastomosis were performed during thyroid cancer extirpation. However, preserving the RLN only in patients whose MACIS scores were ≤ 6.99.

### Supplementary Information

Below is the link to the electronic supplementary material.

Supplementary file1 (DOCX 13 KB)

## Data Availability

All data generated or analysed during this study are included in this published article.
