# Supplementary material for: New strategy for intraoperative phonosurgical management of recurrent laryngeal nerve infiltrated by thyroid carcinoma
Source: Langenbecks Arch Surg. 2024 Apr 27;409(1):138. doi: 10.1007/s00423-024-03323-x (PMC11055930; doi:10.1007/s00423-024-03323-x)
Supplement: Supplementary file 1 — Supplementary file1 (DOCX 13 KB) [file 423_2024_3323_MOESM1_ESM.docx]

**Additional Files**

Table S1. MACIS score and survival.

| Score | Cause-specific Survival (%) |
| --- | --- |
| ＜6 | 99 |
| 6-6.99 | 89 |
| 7-7.99 | 56 |
| ＞8 | 24 |
